# Assessment of Body Mass Index (BMI) in Patients With Beta Thalassemia Major (β-TM): A Hospital-Based Study

**DOI:** 10.7759/cureus.116341

**Published:** 2026-09-16

**Authors:** Divya Gupta, Onkar Awadhiya, Ashok Kumar, Jagdamba Dixit

**Affiliations:** 1 Biochemistry, People's College of Medical Sciences and Research Centre, Bhopal, IND; 2 Internal Medicine, All India Institute of Medical Sciences, Nagpur, IND; 3 Biochemistry, All India Institute of Medical Sciences, Bhopal, IND; 4 Pediatrics, People’s College of Medical Sciences and Research Centre, Bhopal, IND

**Keywords:** blood transfusion, bmi, ferritin, iron overload, 𝛽-tm

## Abstract

Objective

We assessed the body mass index (BMI) and its association in patients with β-thalassemia major (β-TM).

Materials and methods

This hospital-based study was conducted following approval from the Institutional Ethics Committee. All participants underwent a detailed clinical evaluation by a pediatrician, and diagnosis of *β*-TM was established using hemoglobin (Hb) electrophoresis.

Statistical analysis

Clinico-pathological data were compiled using Microsoft Excel (Microsoft Corp., Redmond, Washington, United States) and analyzed with IBM SPSS Statistics for Windows, version 21 (released 2012; IBM Corp., Armonk, New York, United States) for statistical analysis. Data collected from patients with *β*-TM were analyzed using descriptive statistical methods. Shapiro-Wilk test was used to assess normality of data distribution. Categorical variables were compared between subgroups using chi-square test or Fisher’s exact test, wherever appropriate. For continuous variables with normal distribution, mean values were compared using the student’s t-test.

Results

We included 35 patients with 𝛽-TM in our study. They were classified into three age groups: Group I included 13 patients (9 males (M) and 4 females (F)) aged 06-60 months, Group II comprised 14 patients (9M and 5F) aged 61-120 months, and Group III consisted of 8 patients (4M and 4F) aged 121-156 months.. An analysis of underweight (<5th) percentile reported 2M (22.22%)/2F (50.0%), 2M (22.22%)/0F (0%), and 2M (50%)/2F (50%) in groups I, II, and III, respectively. We did not find overweight and obese patients in our study. The line diagram of age and height showed a significant positive correlation, whereas line diagram of BMI and height and BMI and blood transfusion (BT) numbers showed an insignificant correlation in patients with 𝛽-TM.

Conclusion

Early assessment of BMI is crucial to identify nutritional abnormalities and prevent potential complications in patients with β-TM. Patients with 𝛽-TM who meet the criteria for underweight need proper routine checkups; appropriate lifestyle modifications may help improve their quality of life.

## Introduction

𝛽-Thalassemia major (𝛽-TM) is an autosomal recessive inherited disorder characterized by reduced or absent synthesis of β-globin chains relative to α-globin chains, causing an imbalance in globin production and resulting in chronic hemolytic anemia. Globally, an estimated 300,000-400,000 infants are born annually with hemoglobin (Hb) disorders, among whom approximately 23,000 cases are diagnosed with thalassemia major, the most severe form of thalassemia [1].

Thalassemia is distributed worldwide, predominantly concentrated in regions known as the “thalassemia belt,” including the Mediterranean, Middle East, Africa, and South and Southeast Asia; migration from countries such as Italy, Greece, Pakistan, Iran, and India has contributed to an increased prevalence of thalassemia in countries like the United States [2-4]. 𝛽-thalassemia is classified into three categories: 𝛽-TM, which is transfusion-dependent; 𝛽-thalassemia intermedia, which is non-transfusion-dependent (NTDT); and 𝛽-thalassemia carrier status (𝛽-thalassemia minor).

In India, 𝛽-thalassemia is widely prevalent, with approximately 10,000-12,000 infants born each year with 𝛽-TM. It is reported that approximately 42 million 𝛽-thalassemia carriers are present with an average frequency of 3-4% [5,6]. An increased prevalence has been observed in certain Indian communities, including Sindhis, Punjabis, Gujaratis, Bengalis, Mahars, Kolis, Saraswats, Lohanas, and Gaurs [5].

In patients with 𝛽-TM, erythropoiesis leads to premature destruction of erythrocytes, resulting in chronic anemia that is accompanied by extramedullary hematopoiesis (EH) and bone marrow expansion (BME) [7]. As a result of ineffective erythropoiesis, patients with 𝛽-TM depend on lifelong regular blood transfusions (BTs) to keep Hb levels within the range of 9-10.5 g/dL, whereas patients with NTDT typically require only occasional transfusions. Regular BTs result in excessive iron accumulation and hemosiderosis, leading to toxic effects in patients with β-TM [8].

Patients with β-TM commonly develop various complications, such as impaired physical growth, developmental delays, malnutrition, infections acquired through repeated transfusions, organ damage involving the liver, heart, and endocrine glands, ultimately leading to higher morbidity and mortality [7,9-13]. Management and treatment strategies for β-TM comprise regular BTs, iron chelation therapy for controlling iron accumulation, hydroxyurea therapy, splenectomy, enhancement of fetal hemoglobin (HbF) synthesis, and bone marrow or hematopoietic stem cell transplantation [14-16].

Growth impairment is influenced by nutritional imbalances, endocrine abnormalities, and the adverse effects of chelation therapy. Growth disturbance is a major clinical issue in patients with 𝛽-TM. The present study was undertaken to assess BMI and examine its association in patients with β-TM.

## Materials and methods

Patients and clinical examination

This hospital-based study was conducted following approval from the Institutional Ethics Committee. Patients with β-TM were recruited between July and December 2023 at the Departments of Biochemistry and Pediatrics of the institute. This hospital-based pilot study included 35 patients with β-TM aged 13 months to 13 years (13-156 months) who were undergoing regular BT and receiving iron chelation therapy at the institute.

All participants underwent a detailed clinical evaluation by a pediatrician, and diagnosis of β-TM was established using Hb electrophoresis. Clinical details were recorded from the patient’s data sheets (such as date of enrollment, date at the time of diagnosis, details of hematological parameters, total number of BT till date, Hb value, height, weight, and pathological details as per the patient’s proforma). Patients diagnosed with β-TM were excluded if they were younger than 13 months or older than 13 years (156 months) during the study period, had undergone bone marrow transplantation (BMT), were HIV positive, had received high-dose steroid therapy for two weeks or longer, or had any other chronic illness such as tuberculosis, malignancy, or inflammatory bone/joint disorders. Enrolment of subjects was carried out after obtaining informed written consent from the parents, along with verbal assent from patients with β-TM.

Statistical analysis

Clinico-pathological data were compiled using Microsoft Excel (Microsoft Corp., Redmond, Washington, United States) and analyzed with IBM SPSS Statistics for Windows, version 21 (released 2012; IBM Corp., Armonk, New York, United States) for statistical analysis. Data collected from patients with β-TM were analyzed using descriptive statistical methods. Shapiro-Wilk test was used to assess normality of data distribution. Categorical variables were compared between subgroups using the chi-square test or Fisher’s exact test, wherever appropriate. For continuous variables with normal distribution, mean values were compared using the Student’s t-test.

BMI measurement

BMI was calculated using the Centers for Disease Control and Prevention (CDC) Child and Teen BMI Calculator [17], and BMI-for-age percentile categories were interpreted according to the CDC Child and Teen BMI Categories [18] for children and adolescents aged 2-19 years [19]. It was determined using the patient’s height and weight, which were recorded using a calibrated scale in the hospital examination room. BMI was calculated as weight (kg) divided by height squared (m²), and its clinical interpretation was based on BMI percentile. BMI percentile criteria and their clinical interpretation are shown in Table 1.

**Table 1:** BMI percentile criteria and their clinical interpretation of patients with 𝛽-TM BMI percentile criteria were obtained from CDC BMI charts available at http://www.cdc.gov/growthcharts/clinical_charts.htm) [18]. BMI: body mass index; CDC: Centers for Disease Control and Prevention

BMI for age-weight status categories	
BMI percentile	Clinical interpretation
Less than 5^th^ percentile	Underweight
5^th^ percentile to less than 85^th^ percentile	Healthy weight
Less than 85^th^ percentile to less than 95^th^ percentile	Overweight
Equal to or more than the 95^th^ percentile	Obese

## Results

The study included 35 patients with β-TM. The mean age of the patients was 86.17 ± 47.63 months, with an age range of 13-156 months. The clinico-pathological features of patients with β-TM were as follows: 32 (91.4%) had facial dysmorphism, 30 (85.7%) had splenomegaly, 29 (82.8%) had lethargy, 23 (65.7%) had fatigability, 20 (57.1%) had breathlessness at rest, 6 (17.1%) had chest pain, 7 (20.0%) had joint pain, 7 (20.0%) had jaundice, 4 (11.4%) had back pain, 3 (8.6%) had bone pain, 3 (8.6%) had fracture, and 1 (2.9%) had gall stone. The remaining clinico-pathological features, including a history of diabetes, thyroid disorder, hypothyroidism, kyphosis, scoliosis, lordosis, genu varum, genu velgum, and skin pigmentation, were absent (Table 2).

**Table 2:** Clinico-pathological features of patients with 𝛽-TM (N = 35) 𝛽-TM: beta thalassemia major

Clinico-pathological features	Number	Frequency (%)
Facial dysmorphism	32	91.4
Splenomegaly	30	85.7
Lethargy	29	82.8
Fatigability	23	65.7
Breathlessness at rest	20	57.1
Pain in joints	7	20.0
Jaundice	7	20.0
Chest pain	6	17.1
Back pain	4	11.4
Bone pain	3	8.6
Fracture	3	8.6
Gall stone	1	2.9
History of diabetes	0	0
Thyroid disorder	0	0
Hypothyroidism	0	0
Kyphosis	0	0
Scoliosis	0	0
Lordosis	0	0
Genu varum	0	0
Genu velgum	0	0
Skin pigmentation	0	0

In our study, patients with *β*-TM were classified into three age groups: Group I included 13 patients (9 males (M) and 4 females (F)) aged 06-60 months, Group II comprised 14 patients (9M and 5F) aged 61-120 months, and Group III consisted of 8 patients (4M and 4F) aged 121-156 months. Clinical, hematological, transfusion-related, and anthropometric parameters of patients with β-TM are presented in Tables 3-4.

**Table 3:** Clinical, hematological, transfusion-related, and anthropometric parameters of patients with 𝛽-TM Mean and standard deviation values of clinical, hematological, transfusion-related, and anthropometric parameters of patients with β-TM were calculated/verified using the standard deviation calculator [17]. 𝛽-TM: beta thalassemia major; BMI: body mass index; BT: blood transfusion; Hb: hemoglobin; SD: standard deviation

Parameters	Mean value	Range		SD
		Min.	Max.	
Age in months	86.17	13 months	156 months	47.63
First BT age (months)	12.21	1 month	46 months	11.90
Hb value before first BT	5.55	2.3	8.9	1.45
Hb value currently available	7.40	2.4	11.0	1.65
Height (in cm)	112.97	75.0	144.0	20.94
Upper segment (US height (in cm))	54.68	39.0	70.0	8.20
Lower segment (LS)	58.28	33.0	83.0	14.06
US:LS ratio	0.952	0.12	1.48	0.22
Weight (kg)	19.54	9.38	35.14	7.10
BMI (kg/m^2^)	14.86	12.7	17.8	1.29
Total no. of BT	67.77	1.00	360.0	66.88

**Table 4:** Clinical, hematological, transfusion-related, and anthropometric parameters according to gender in patients with 𝛽-TM 𝛽-TM: beta thalassemia major; BT: blood transfusion; SD: standard deviation; BMI: body mass index

Parameters	Male (22) 62.85%		Female (13) 37.14%	
	Mean	SD	Mean	SD
Age in months	81.95	46.93	93.30	49.86
First BT age (months)	12.81	12.61	11.19	11.03
Hb value before first BT	5.80	1.37	5.12	1.53
Hb value (currently available)	7.60	1.82	7.06	1.31
Height (in cm)	110.63	20.39	116.92	22.09
Upper segment (US)	54.59	7.84	54.84	9.10
Lower segment (LS)	56.04	13.79	62.07	14.23
US:LS ratio	0.97	0.25	0.921	0.149
Weight (in kg)	19.50	7.89	20.97	8.29
BMI (kg/m^2^)	14.93	1.16	14.74	1.53
Available number of BT	73.27	79.24	58.46	39.21

We also calculated the mean value (https://www.calculator.net/standard-deviation-calculator.html) of the age when the diagnosis was made, 12.86 months, and the mean age of the first BT, 12.22 months (there was variation in the age at diagnosis and the age at which the first BT was received). The standard deviation (SD) values (Table 3) were calculated from the individual patient-level data using the SD calculator available at Calculator.net. The calculated SD values were checked against the values presented in Table 3 and were found to be consistent. Therefore, the SD values reported in Table 3 were confirmed.

The mean pre-transfusion Hb level was determined from the Hb value recorded at diagnosis (5.5 g/dL), while the average Hb level measured at enrollment is shown in Table 3. The mean values of clinical, hematological, transfusion-related, and anthropometric parameters according to sex are presented in Table 4.

Body mass index

The BMI distribution of patients with β-TM according to gender and age is shown in Tables 5-6. We also plotted the line diagrams of BMI and height, BMI and BT numbers, and age and height, shown in Figures 1-3, respectively.

**Table 5:** BMI distribution of patients with 𝛽-TM according to gender M: Male; F: Female; N: number of patients; BMI: body mass index; 𝛽-TM: β-thalassemia major

Patients with 𝛽-TM		BMI percentile (%)			
		< 5^th ^Underweight 10 (28.6)	5^th^ to 85^th^ Healthy 25 (71.4)	85^th^ to 95^th^ Overweight	>95^th^ Obese
Gender	Male N = 22 (62.85%)	6 (27.2)	16 (72.2)	-	-
	Female N = 13 (37.14%)	4 (30.7)	9 (69.2)	-	-

**Table 6:** BMI distribution of patients with 𝛽-TM according to age groups Group I: 13-60 months; Group II: 61-120 months; Group III: 121-156 months; N: number of patients; Freq.: frequency; BMI: body mass index; 𝛽-TM: β-thalassemia major

Patients with 𝛽-TM		BMI percentile (%)							
		< 5^th ^Underweight 10 (28.6%)		5^th^ to 85^th^ Healthy 25 (71.4%)		85^th^ to 95^th^ Overweight		>95^th^ Obese	
Age (months)	Male (N = 22)	N = 6	Freq. %	N = 16	Freq. %	N	Freq.%	N	Freq.%
	Group I (N = 9)	2 M	22.22	7 M	77.77	-	-	-	-
	Group II (N = 9)	2 M	22.22	7 M	77.77	-	-	-	-
	Group III (N = 4)	2 M	50.0	2 M	50.0	-	-	-	-
	Female (N = 13)	N = 4	Freq. %	N = 9	Freq. %	N	Freq.%	N	Freq.%
	Group I (N = 4)	2 F	50.0	2 F	50.0	-	-	-	-
	Group II (N = 5)	0	0	5 F	100	-	-	-	-
	Group III (N = 4)	2 F	50.0	2 F	50.0	-	-	-	-

**Figure 1:**
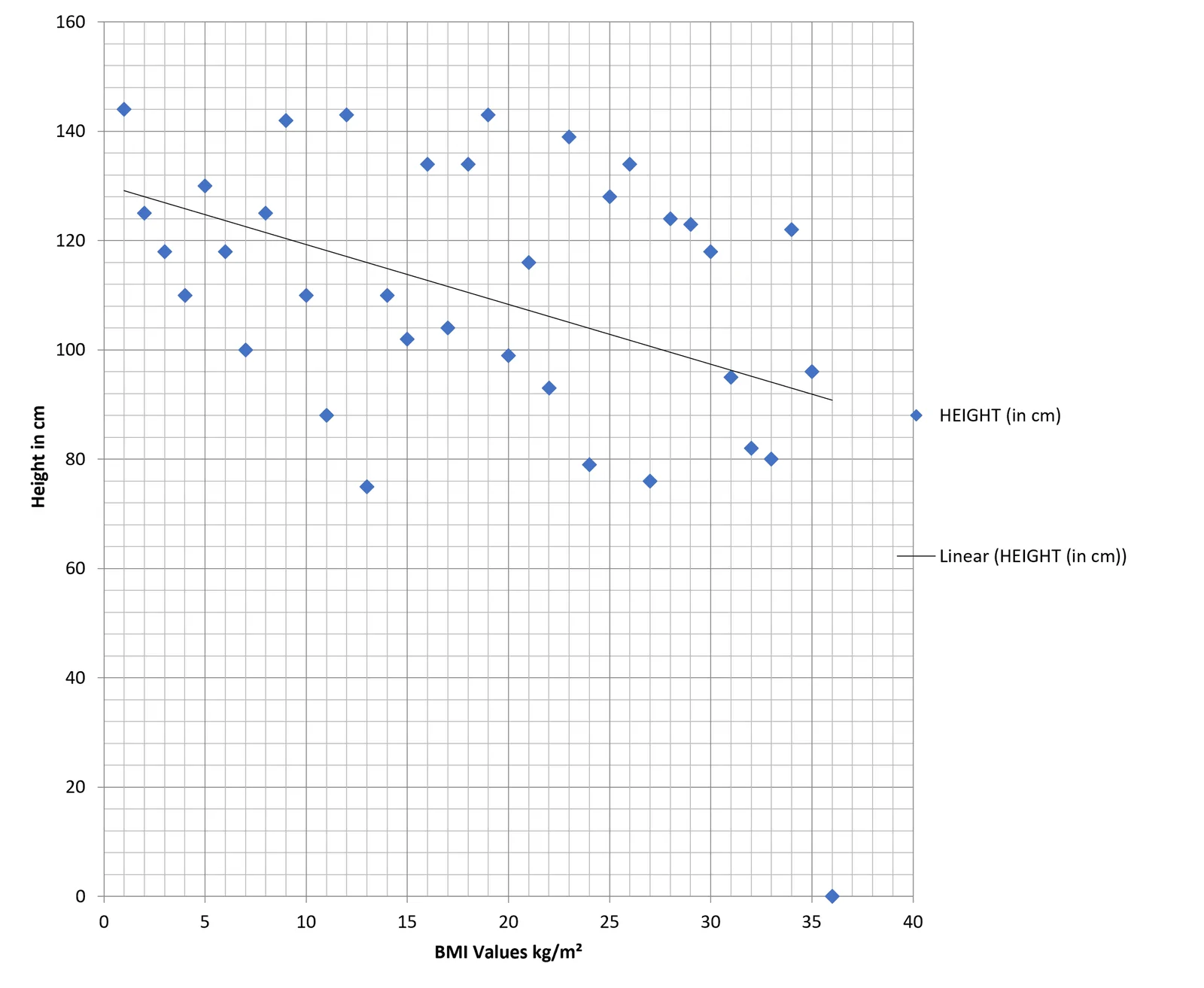
Line diagram of BMI and height of patients with 𝛽-thalassemia major The figure was created using Microsoft Excel (version 16; Microsoft Corp., Redmond, Washington, United States). BMI: body mass index

**Figure 2:**
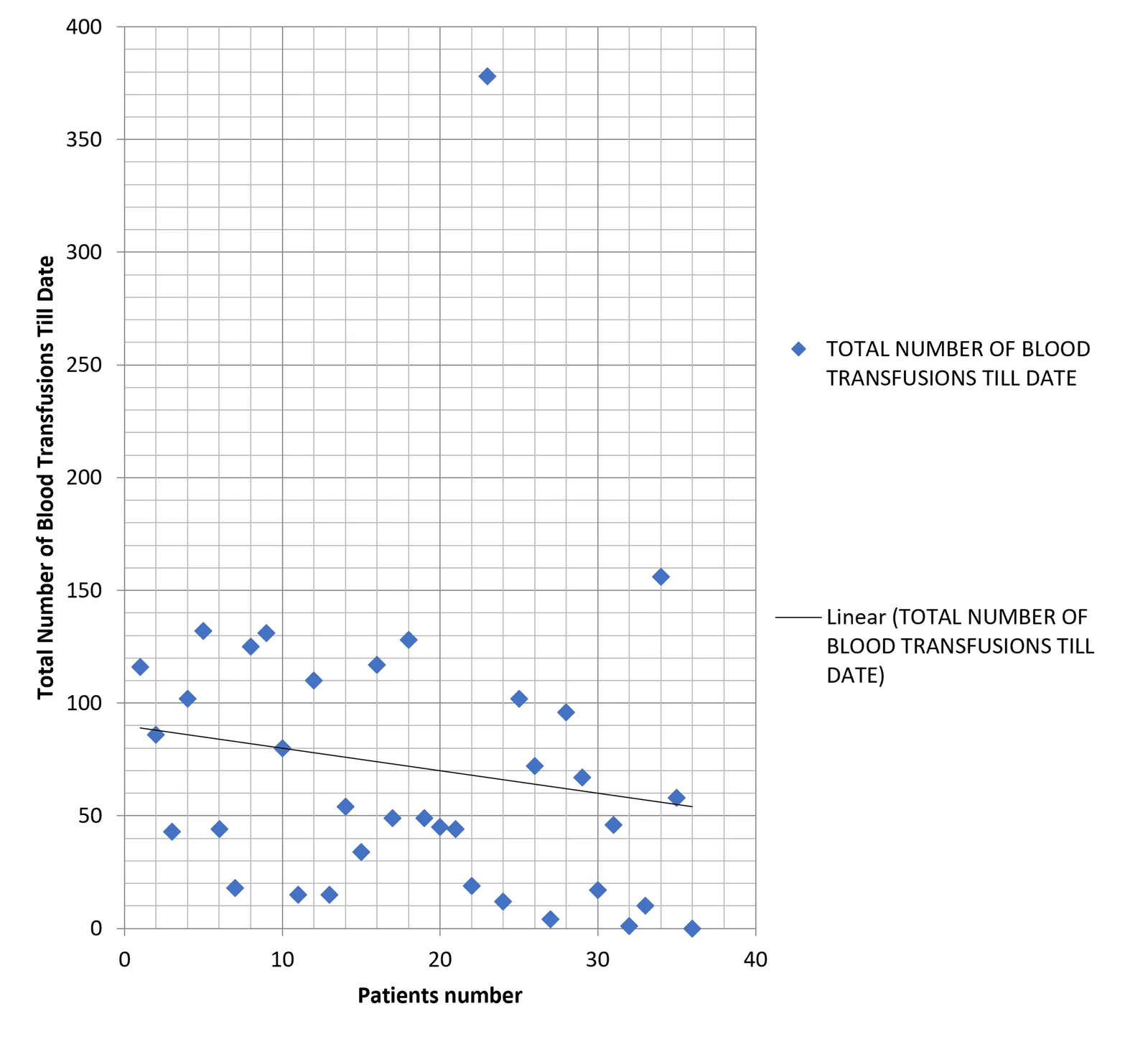
Line diagram of BMI and BT numbers of patients with 𝛽-thalassemia major The figure was created using Microsoft Excel (version 16; Microsoft Corp., Redmond, Washington, United States). BMI: body mass index; BT: blood transfusion

**Figure 3:**
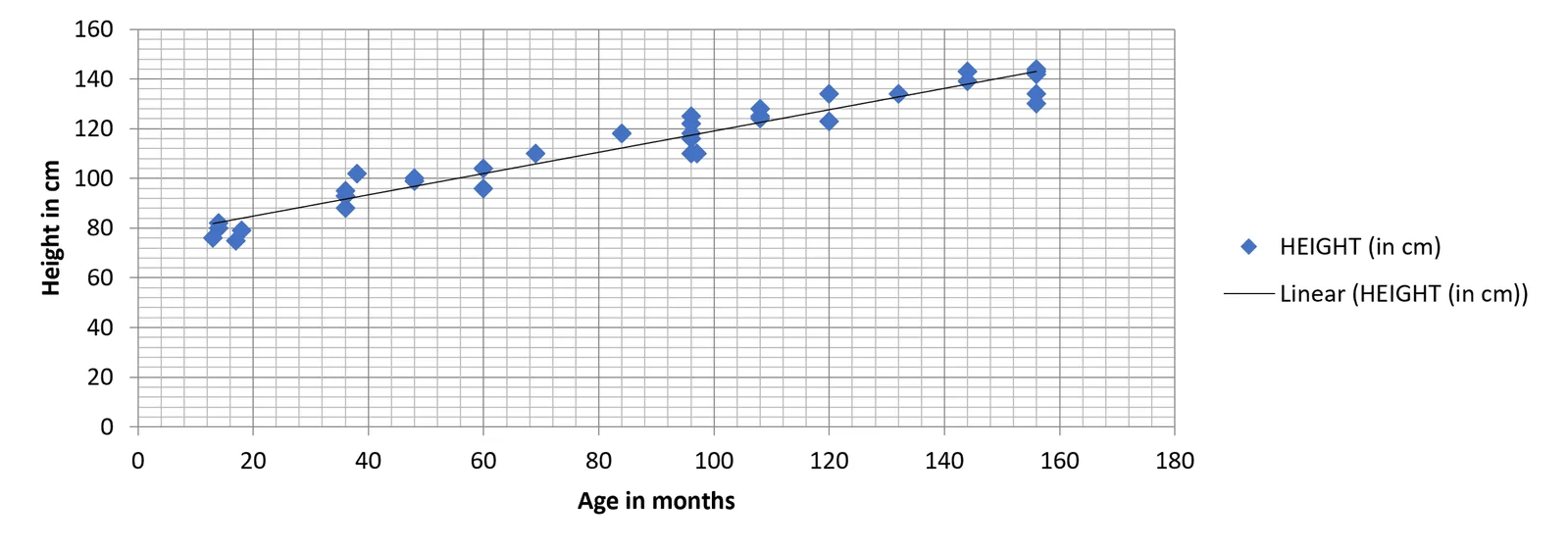
Line diagram of age and height of patients with 𝛽-thalassemia major The figure was created using Microsoft Excel (version 16; Microsoft Corp., Redmond, Washington, United States).

Figure 1 presents a scatter plot of height measurements among the 35 children with β-TM. Each blue diamond represents the height of an individual patient. The heights show considerable variability, ranging approximately from 75 cm to 144 cm, with most observations falling between 100 and 140 cm. The overall mean height in the study population was 112.97 ± 20.94 cm.

Figure 2 demonstrates the relationship between BMI and the number of BTs; the X-axis is labeled as BMI (kg/m²), and the Y-axis represents the total number of BTs to date.

Figure 2 shows the distribution of the total number of BTs received by patients with β-TM. The scatter plot demonstrates considerable inter-individual variation in the cumulative number of BTs. Most patients had received fewer than 150 transfusions, while a few patients had substantially higher transfusion requirements, with one patient having received approximately 380 transfusions.

Figure 3 demonstrates a clear positive linear relationship between age X-axis and height Y-axis among the studied patients with β-TM. The X-axis is labeled as BMI (kg/m²), and the Y-axis represents the total number of BTs to date. Height increased progressively with age, although some variability in height was observed among patients of comparable age. This finding reflects the age-dependent increase in stature, while the individual variation may be related to the effects of chronic disease, nutritional status, anemia, iron overload, and other growth-affecting factors in β-TM. The line diagram of age and height of patients with 𝛽-TM shows the significant correlation (Figure 3). Height increased with increasing age; however, the correlations between BMI and height and between BMI and the total number of BTs were statistically insignificant. BMI percentile criteria and clinical interpretation were determined as given in Table 1.

BMI percentile shows that in 35 patients with 𝛽-TM, 10 (28.6%) were underweight (<5th) and 25 (71.4%) were reported as healthy weight (5th percentile to <85th percentile) (Table 5). Out of 35 patients with 𝛽-TM (22 M and 13 F), 16 (72.2%) of male patients and 9 (69.2%) of female patients had healthy weight, and 6 (27.2%) male patients and 4 (30.7%) female patients (Table 6) were reported as <5th percentile (underweight). Among Groups I, II, and III, comprising 13, 14, and 8 patients, respectively, underweight status (<5th percentile) was observed in 4 (30.7%), 2 (14.2%), and 4 (50.0%) patients (2M/2F (30.7%), 2M (14.2%), and 2M/2F (50%)), respectively, as shown in Table 5. The overall mean BMI was 14.86 kg/m². No patients were classified as overweight or obese in our study.

## Discussion

Patients with 𝛽-TM are prone to various complications, especially growth and developmental disturbances, malnutrition, transfusion-acquired infections, and tissue injury involving the liver, heart, and endocrine system, which collectively lead to higher morbidity and mortality rates. The present study primarily describes BMI distribution among patients with β-TM, and larger studies with adequate subgroup analyses are required to evaluate associations between BMI and specific clinical complications. BMI of patients with 𝛽-TM plays a significant role in their growth and development; therefore, it shows an association with patients with 𝛽-TM. BMI is used to classify individuals as underweight, healthy weight, overweight, or obese. Available studies indicate that the majority of the patients with 𝛽-TM were underweight. Salih et al. [20] reported that approximately two-thirds (60%) of patients with thalassemia were underweight, while the remainder had normal weight or were overweight. In contrast, our study showed that 28.5% of cases were underweight and 71.4% of patients were healthy. A study by Lidoriki et al. [21] reported that, based on BMI values, 70.1% of patients had normal weight, 7.5% were underweight, and 22.4% were overweight or obese. The authors also noted that their study results were similar to those of a previously published study, which found that the prevalence of underweight patients was 5.2%, the prevalence of overweight/obese patients was 26.5%, and approximately 70% of patients were classified as normal [22].

The present hospital-based pilot study included 35 patients with β-TM aged 13 months to 13 years (13-156 months), whereas several previous studies have included different age groups, larger populations, or adults. These methodological and demographic differences may partly explain the variation in the prevalence of underweight, healthy weight, overweight, and obesity among studies [22]. Sheikh et al. [23] reported that children with 𝛽-TM were aged 2-16 years, with a mean BMI of 17.9 kg/m². Among the study participants, 179 (58.69%) were underweight, 9 (2.95%) had normal weight, 10 (3.28%) were overweight, and 7 (2.30%) were obese. It is reported in several studies that growth impairment may be attributed to the toxic effects of deferoxamine, excessive iron deposition in tissues, and impairment of iron-dependent enzymes involved in collagen modification [24].

We recorded that most patients with β-TM from rural areas registered in the study had weak economic conditions and also faced nutritional issues. In our study, approximately 27-31% of patients with 𝛽-TM were underweight, and 70% were reported as having a healthy BMI.

Underweight may be associated with numerous health problems; therefore, early detection of these abnormalities is crucial to prevent further complications in patients with 𝛽-TM. Several studies have demonstrated that the majority of patients with 𝛽-TM tend to be underweight. Moreover, both undernutrition and obesity may contribute to adverse health outcomes in patients with 𝛽-TM, including an increased the risk of coronary heart disease. Therefore, early detection of these abnormalities is essential to prevent potential adverse outcomes in patients with β-TM.

A study from India by Biswas et al. [25] reported that 48.2% of children with thalassemia were malnourished, with a mean BMI of 13.9 ± 1.6 kg/m². The authors also reported that malnourished children were classified as underweight. These findings are consistent with our observations, as none of the children in our study were classified as overweight or obese. A study conducted in Thailand by Tanphaichitr et al. reported a 74.5% prevalence of protein-energy malnutrition among thalassemic children, based on a weight-for-age value below 90% of the standard [26]. Growth impairment in β-TM is multifactorial.

Patients with β-TM may have inadequate dietary intake of several micronutrients, including vitamins A, D, E, and K, folate, calcium, phosphorus, zinc, and magnesium, relative to the recommended dietary allowances. These nutritional inadequacies may contribute to poor nutritional status and impaired growth. Growth impairment in β-TM is multifactorial and may be associated with chronic anemia, iron overload, elevated serum ferritin levels, inadequate iron-chelation therapy, insufficient dietary intake, and endocrine dysfunction. Iron overload and chronic disease may adversely affect endocrine organs and contribute to abnormalities in growth and other endocrine parameters. Underweight patients with β-TM may have an increased risk of morbidity and mortality. Therefore, greater attention should be given to underweight patients, ensuring they receive proper routine checkups, and appropriate lifestyle modifications may help improve the quality of life for those with 𝛽-TM. We observed and recorded that most patients with 𝛽-TM registered in the study were from rural areas. Limited socioeconomic resources may have contributed to nutritional imbalances among these patients. The major limitation of our study was the relatively small sample size. Larger multicenter studies are required to validate these findings. In addition, serum ferritin testing was not routinely available at government-funded primary health centers, which represented an additional financial burden for patients. Larger study samples are needed to validate the results and improve the quality of care and prevent complications in patients with 𝛽-TM.

## Conclusions

Being underweight may be associated with numerous health problems; therefore, early detection of these abnormalities is crucial to prevent further complications in patients with 𝛽-TM. Therefore, early assessment and regular monitoring of BMI are important for identifying nutritional abnormalities and reducing the risk of subsequent complications in patients with β-TM. Several studies have reported a high prevalence of underweight among individuals with β-TM. Patients who meet the criteria for underweight should undergo regular nutritional and clinical assessment, along with appropriate dietary and lifestyle interventions, to improve their nutritional status and overall quality of life.
